# Learning collaboration at the primary-secondary care interface: a dual-method study to define design principles for interventions in postgraduate training programmes

**DOI:** 10.1186/s12909-023-04254-9

**Published:** 2023-05-03

**Authors:** Marijn Janssen, Cornelia R.M.G. Fluit, Roel R. Lubbers, Sylvia A. Cornelissen, Jacqueline de Graaf, Nynke D. Scherpbier

**Affiliations:** 1 Department of Internal Medicine Nijmegen, Radboud University Medical Center, Geert Grooteplein Zuid 10, PO box 9101, postal route 463, Nijmegen, 6500 HB The Netherlands; 2 Radboudumc Health Academy, Radboud University Medical Center, Nijmegen, The Netherlands; 3 Department of General Practice and Elderly Care Medicine, University Medical Center Groningen, Groningen, The Netherlands; 4 Obstetrics and Gynaecology, Radboud University Medical Center, Nijmegen, The Netherlands; 5 Department Primary and Community Care, Radboud University Medical Center, Nijmegen, The Netherlands

**Keywords:** Primary-secondary care collaboration, Postgraduate training programmes, Medical education design

## Abstract

**Background:**

Collaboration between primary and secondary care (PSCC) is important to provide patient-centered care. Postgraduate training programmes should provide training to learn PSCC. With a design based research (DBR) approach design principles can be formulated for designing effective interventions in specific contexts. The aim of this study is to determine design principles for interventions aimed to learn PSCC in postgraduate training programmes.

**Methods:**

DBR is characterised by multi-method studies. We started with a literature review on learning collaboration between healthcare professionals from different disciplines within the same profession (intraprofessional) to extract preliminary design principles. These were used to inform and feed group discussions among stakeholders: trainees, supervisors and educationalists in primary and secondary care. Discussions were audiotaped, transcribed and analysed using thematic analysis to formulate design principles.

**Results:**

Eight articles were included in the review. We identified four preliminary principles to consider in the design of interventions: participatory design, work process involvement, personalised education and role models. We conducted three group discussions with in total eighteen participants. We formulated three design principles specific for learning PSCC in postgraduate training programmes: (1) The importance of interaction, being able to engage in a learning dialogue. (2) Facilitate that the learning dialogue concerns collaboration. (3) Create a workplace that facilitates engagement in a learning dialogue. In the last design principle we distinguished five subcategories: intervention emphasises the urge for PSCC and is based on daily practice, the presence of role models, the work context creates time for learning PSCC, learning PSCC is formalised in curricula and the presence of a safe learning environment.

**Conclusion:**

This article describes design principles for interventions in postgraduate training programmes with the aim to learn PSCC. Interaction is key in learning PSCC. This interaction should concern collaborative issues. Furthermore, it is essential to include the workplace in the intervention and make adjacent changes in the workplace when implementing interventions. The knowledge gathered in this study can be used to design interventions for learning PSCC. Evaluation of these interventions is needed to acquire more knowledge and adjust design principles when necessary.

**Supplementary Information:**

The online version contains supplementary material available at 10.1186/s12909-023-04254-9.

## Introduction

In several countries primary care and secondary care play an important role in health care organisation. The aim of primary care is providing access and use of health services whenever necessary, comprehensiveness, coordination and continuity of care [1]. Secondary care mostly takes place in hospitals, is more specialised and is only accessible through referral. Collaboration between primary and secondary care is a prerequisite for the provision of sustainable patient-centered care in a time where ageing, multimorbidity and rising complexity of care are major problems [2–4]. Primary-secondary care collaboration (PSCC) does not always go smoothly [5, 6]. Poor collaboration can lead to patients having reduced confidence in care providers and increasing anxiety [7]. Research on discharge processes shows that transitions between primary and secondary care are associated with a substantial number of medical errors [8]. Johnston et al. suggested that conflicts at the primary-secondary care interface originate from two different ways of knowing medicine. With secondary care representing the ‘neutral’ scientific method and primary care utilising a more narrative patient-centred approach [9]. Doctors play an important role in PSCC and optimisation of their collaborative behaviour could lead to improved collaboration. This means doctors should be adequately trained for PSCC.

A specific moment of interest for this training is during postgraduate training programmes. During their undergraduate medical education students follow the same curriculum. After graduating most doctors choose to specialise and start with separate training programmes. During this training primary and secondary care doctors follow mainly monodisciplinary based education programmes and socialisation into their own group of professionals takes place [9–11]. To prevent that this separate socialisation hampers PSCC, this is the time special attention towards learning PSCC is needed.

Primary and secondary care trainees acknowledge the importance of learning about PSCC but mention that explicit education in such collaboration is not provided. They mostly learn informally through interaction in the patient-care context [12, 13]. To make such interaction more meaningful and create valuable learning opportunities, postgraduate training programmes should pay more explicit attention to learning about PSCC [14].

The main way of acquiring new knowledge, skills and attitudes in postgraduate training programmes is through workplace based learning [13]. Although workplace based learning is a very effective way of acquiring knowledge and skills needed in medical practice, workplace learning also has disadvantages including lack of explicit learning goals, lack of access to the right activities and lack of time or support for reflection, thinking and interpreting [15]. Workplace based educational interventions can support trainees to make more meaning out of their work related activities and use them as learning opportunities to learn PSCC. Although competencies for PSCC have been defined [16, 17], and design principles for PSCC in a hospital setting have been developed [18], there is no evidence in how to design education for PSCC in a broader setting including primary care.

A research method suitable for the investigation of complex educational practices is design based research (DBR) [19, 20]. One of the goals of DBR is the formation of design principles that specify which characteristics an intervention should have to succeed in a specific context [19, 21]. Determination of these principles can inform further development of educational interventions. Therefore the aim of our study was to define design principles for educational interventions to learn PSCC in postgraduate training programmes. This resulted in the following research question: “which design principles can be defined for educational interventions to learn PSCC in postgraduate training programmes?”

## Methods

The development of educational interventions in postgraduate training programmes faces challenges. In workplace learning the role of the context, the workplace, is very important. However, this context is complex and sensitive to changes [14]. DBR attempts to bring theory and practice closer together through iterative analysis, design, development, and implementation, based on collaboration among researchers and practitioners in real-world settings [19, 20]. Four phases can be distinguished in DBR. In phase 1 practical problems are analysed by researchers and practitioners. In Phase 2 draft design principles and solutions are formulated. In phase 3 solutions are tested and refined in practice. And last, phase 4 refers to reflection to produce design principles and enhance solution implementation [22]. In this study we aimed to define draft design principles for learning PSCC in postgraduate training programmes of general practitioners (GPs) and hospital based medical specialists (MSs). Design principles can refer to characteristics of the intervention (content, form), or how it should be developed and implemented [23]. We combined two research methods. We started with a literature review and used the results to inform group discussions among stakeholders.

In 2023 we performed an additional literature search (literature from 2017 to 2023) and subsequent analysis to see if our findings are still in line with current knowledge. We will discuss this in the results section after the results of the group discussions.

### Research team

The research team consisted of six researchers with different backgrounds; an educationalist (CF), a GP (NS), MS in internal medicine (JG), MS trainee in internal medicine and PhD student (MJ) and two medical students (RL and SC). CF, NS, JG and MJ had experience in conducting qualitative research. They had experience in interviewing, leading focus groups, content and thematic analysis. During the whole process, the research team worked closely together to make sure different points of view were taken into account.

### Research context: Dutch post graduate training programmes of general practice and hospital based medical specialty training

Postgraduate training programmes and their duration vary per specialty and per trainee. Most GP trainees follow a three-year training programme. In years one and three of GP specialty training, trainees work in general practice. When they work in general practice GP trainees are coached and instructed by one supervisor. GP trainees attend a day-release programme in groups of approximately twelve trainees once weekly. In their second year, GP trainees complete rotations in other care settings, for example emergency rooms, nursing homes and psychiatric outpatient clinics, with different supervisors.

Hospital-based MS training programmes vary from four to six years and mainly take place in academic and non-academic hospitals [24]. Each specialty has their own program director in each hospital. All MSs in a teaching hospital are involved in daily supervision of MS trainees.

### Literature review

In our review we searched for interventions with the aim to improve or learn PSCC in postgraduate training programmes. We used elements from the realist review approach to understand the relationship between the context in which the intervention is applied, the mechanisms by which it works and the outcomes that are produced [25–27]. We started our search with the following terms in various combinations: collaboration, intraprofessional, education, primary care (or general practitioners), secondary care (or specialists) and postgraduate (or residency graduates). This lead to a very low number of eligible studies, therefore we needed to broaden our scope. We decided to expand our inclusion criteria and included intervention studies developed for healthcare professionals with different disciplines within the same profession, instead of only doctors and postgraduate training programmes. Finally, references of included studies were searched for other articles meeting the inclusion criteria (snowball sampling). Our exact search strategy per database is described in appendix 1: Search strategy [28].

**Fig. 1:**
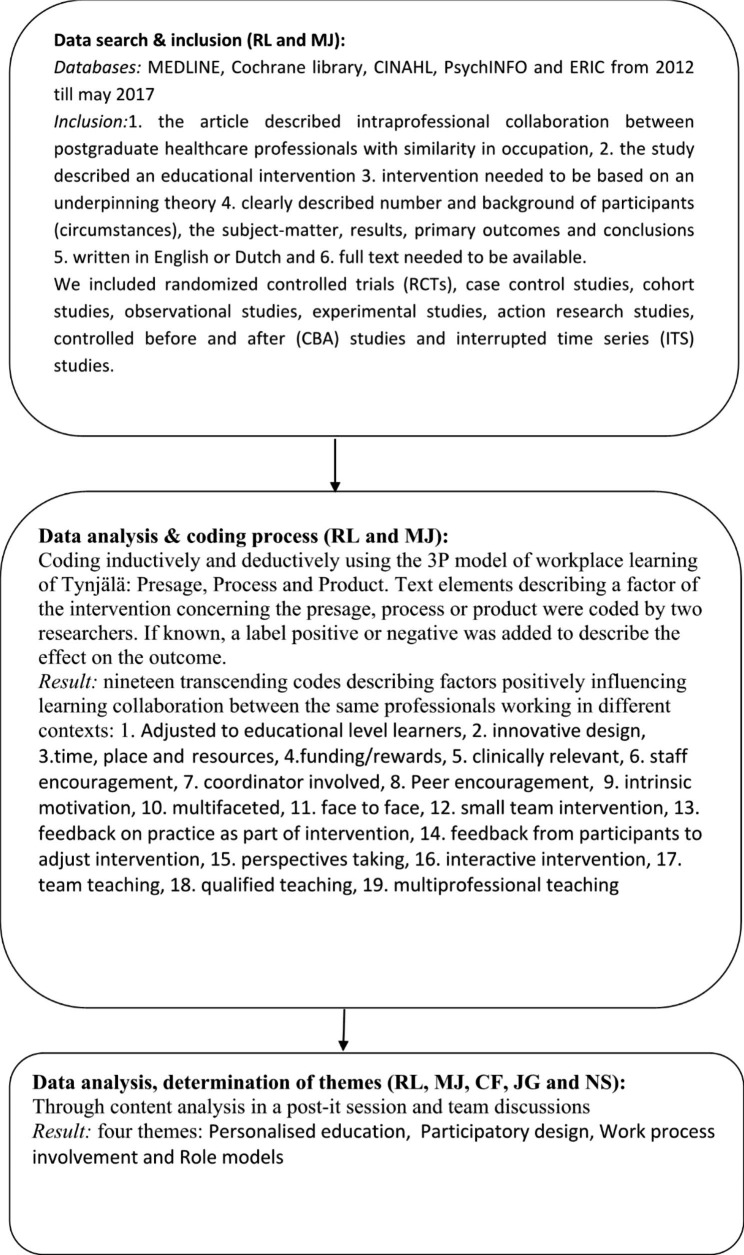
Literature review approach

The 3P (presage, process and product) model by Tynjala was used as a framework for data extraction and analysis [29]. More information on our review approach can be found in Fig. 1.

### Group interviews

Based on the included literature we formulated pre-liminary design principles for interventions to learn intraprofessional collaboration (intraPC). Next, we organised group discussions among stakeholders to make these draft principles more specific for learning PSCC in the context of postgraduate training programmes of doctors [30].

#### Participants

We chose to include primary and secondary care doctors (GPs and MSs) and their trainees (GP trainee and MS trainee) as well as education developers (ED) in our study population. GPs, MSs and their trainees are the people who need to learn PSCC and education developers play a role in the design of education in postgraduate training programmes. We used purposive sampling to select our participants. We aimed to include at least six participants with a maximum of ten participants per group [31]. We composed heterogeneous groups based on care setting (primary or secondary), occupation (doctor or education developer) and experience (trainee or supervisor) to include different perspectives in one group. This way different stakeholders could reflect on other stakeholders’ perspectives. Participants received a gift card of 25 euro for their invested time.

#### Data collection

The group interviews took place at the Radboud Medical University Center in Nijmegen in the Netherlands. We developed a semi-structured interview guide (Appendix 2). An experienced moderator (MJ) led the discussion to ensure that all topics were discussed and all participants were involved in the discussion. An assistant (SC) observed the group interviews and made notes. The interview guide started with questions on general ideas of participants for learning collaboration, followed by more specific questions for learning PSCC in the context of postgraduate training programmes. During a break the moderator and assistant checked which design principles from literature were already discussed upon and after the break we discussed design principles found in literature, which were not addressed previously in the discussion [32]. We performed group interviews until data-saturation was achieved. Data-saturation was defined as “when data collection reveals no new information, and the collected material is redundant”[31].

#### Data analysis

Each interview was recorded on audiotape and transcribed verbatim. The interviews were analysed using qualitative thematic analysis consisting of familiarising with the data, generating initial codes, searching for themes, reviewing potential themes and finally defining and naming themes [33]. ATLAS.ti software version 7.1.4 was used to organise the qualitative data gathered. Working independently, two researchers (MJ and SC) used an inductive approach to select quotations and coded the transcripts. After discussion with other members of the research team (CF, NS and JG), we categorised codes and identified themes [33]. After each interview the data was analysed and, when necessary, changes were made to the interview guide. Reflexive writing on the process of analysis (SC) and group discussions were used to optimise teamwork in the research group and increase trustworthiness [34].

#### Ethical considerations

We conducted our research in line with the principles of the Declaration of Helsinki. Ethical approval from the Dutch research ethics committee (Central Committee on Research Involving Human Subjects (CCMO)[35]) was not required, because our study did not breach the physical or mental integrity of our study population. Our study population was not subjected to actions nor were behavioural rules imposed on them [35]. Participating in the group interviews was voluntarily. Two researchers (SC or MJ) approached the participants, no negative effect of refusal could be expected. We did not put trainees in the same group as their own supervisors. Participants were informed about the aim of the research and the research procedure by e-mail. After reading this information they could decide to participate or to refuse participation. Group interviews were planned with the persons who chose to participate. Before the group interviews started participants gave verbal consent for the interviews and for audiotaping according to the guidelines of the CCMO [35]. The audiotapes were saved in a secure environment and the transcripts were anonymised before analysis took place.

## Results

### Literature review

Eight articles were included in the review [36–43] (appendix 3). We identified four design principles for interventions to learn intraPC: Participatory design, work process involvement, personalised education and role models. Table 1 shows a short description of the design principles and the associated codes.

**Table 1:** Design Principles literature review

Principle	Participatory design	Work process involvement	Personalised education	Role models
**Summary of content**	There is an active participatory role of participants both as content of the intervention as in the implementation and adjustment of the intervention.	Practice or workplace based interventions. Literature showed the importance of support from the work context: creating time, good coordination by people who know the workplace, sufficient funding and encouragement from staff and peers. Intrinsic motivation is enhanced by clinically relevant interventions.	Attractive, innovative interventions adjusted to the learners taking into account differences in education levels of participants.	Intervention is supervised by qualified people with different backgrounds and involves role models from practice.
**Codes**	Multifaceted Face to face Small team intervention Feedback on practice as part of intervention Feedback from participants to adjust intervention Perspectives taking Interactive intervention	Time, place and resources Funding/rewards Clinically relevant Staff encouragement Coordinator involved Peer encouragement Intrinsic motivation	Adjusted to educational level learners Innovative design	Team teaching Qualified teaching Multiprofessional teaching

### Group interviews

Subsequently we conducted three group interviews with a total of eighteen participants between August and October 2017 (Table 2). We identified three design principles for interventions to learn PSCC in postgraduate training programmes, the aim of our study. One principle related to the importance of interaction: engage in a learning dialogue. One to the content of this learning dialogue: facilitate that the dialogue concerns collaboration. And one to the importance of a supportive context of learning: create a workplace that facilitates to engage in a learning dialogue (Table 3).

**Table 2:** Participants and duration of group discussions

	Group interview 1	Group interview 2	Group interview 3	Total
**Participants**	6	7	5	18
**GP trainees (GPT)**	2	2	1	5
**MS trainees (MST)**	1 (internal medicine)	2 (neurology, internal medicine)	1 (urology)	4
**GPs**	0	1	1	2
**MSs**	1 (surgery)	1 (pediatrics)	1 (internal medicine)	3
**Education developers (ED)**	2	1	1	4
**Gender: Male**	1	1	4	6
**Gender: Female**	5	6	1	12
**Duration of group interview**	98 min	104 min	94 min	

### Design principle 1 Interaction: engage in a learning dialogue

One key principle for interventions to learn PSCC was to give GP- and MS trainees the chance to engage in a learning dialogue with each other to create mutual understanding. Interaction was indicated a key factor in learning collaboration. This interaction could be face-to-face but also digitally. Experiencing each other’s work context was emphasised as one of the strongest learning mechanisms, however several less time-consuming possibilities as observation, joint education or telephonic interaction were also seen as promising.

*MST (interview 1): “It would be great for specialty trainees to deliver care in the primary care setting. Not only observing. The experience will be greater. The more emotion, the better. Because you are responsible, you have to make choices… … it would be great for healthcare issues that are both treated in primary and secondary care, like diabetes.”*

*MST (interview 3): “It would be valuable to visit a patient at home after I’ve done an operation and have discharged the patient. ……. to see what happens at home, what questions and incertanties the patient, GP and careteam at home experience. Things they (patients eds.) probably not mention at their outpatient clinic appointment”*.

*ED (interview 2): “That an MS and a GP visit the patient at home, together. Learning collaboration in the context of patient care. That would be very valuable, but also costs a lot of time. I can imagine that instead of a whole day observing, which would be great in my opinion, you could explain to each other how your work day is organised and what problems you face. I know, it is less powerfull than expiriencing, but easier to organise. And than with small steps……”*.

### Design principle 2 facilitate that the learning dialogue concerns collaboration

Participants agreed that in order to learn PSCC interventions should facilitate that the dialogue is about knowledge and skills needed for this collaboration. They named the following topics as important in the dialogue: (1) Each other’s roles, expertise and contexts (2) Discussing or making collaboration agreements, and (3) Reflection on the collaborative process. They mentioned that current contacts between primary and secondary care doctors mainly focus on medical content, which was often valuable, but could be made more valuable when the process of collaboration and each other’s roles would be explicitly addressed as well.

*GPT (interview 3): “If we, GPT and MST, would see patients together, we could learn from each other. …… Every doctor finished two months of a GP internship but then you are not aware of your future specialty. So if you visit a GP during your specialty training and experience how the GP may struggle with a patient with chest pain while as trainee cardiology in the hospital you have your troponines and ECG………………….That way you can learn the considerations a GP makes when the GP refers a patient”*.

*ED (interview 2): “When you called the hospital after you sent in a patient for consultation, do you discuss the referral process and how the collaboration went?” GPT1 “sometimes, when I feel the person on the other side of the phone has time and it feels like a low threshold contact than I ask quickly, “what did you think of the referral and our deliberation?”’ ED “Does that happen often? GPT2: “I think that in practice most of the time we check: did I make the correct diagnosis”*.

### Design principle 3 create a workplace that facilitates engagement in a learning dialogue

In all group interviews the importance of the workplace came forward. It was mentioned that without support from and changes in the work context interventions with the aim to learn PSCC will not succeed. This means that in development and implementation of interventions the workplace should be taken into account as a variable that affects learning PSCC. We distinguished five subcategories.

#### Intervention emphasises the urge for PSCC and is based on daily practice

All participants felt that interventions should take place in or are based on daily practice. Participants emphasised that trainees learn most from situations they recognise in and from daily practice. Participants felt that the need to engage in PSCC is not felt by all colleagues, trainees and supervisors, involved in this collaboration. They felt it was important to feel this need in order to engage in a learning dialogue. Emphasising today’s patient’s expectations and the need for improved work efficiency in interventions and in daily practice could contribute to feeling this need to collaborate and learning this collaboration.

*Moderator (interview 3): “We heard in previous group interviews that it is hard to learn and change when the workplace is not changing. What could help to change daily practice?” GP: “[.] the patient, I think. Today’s patients tell what they want and who they expect to do it. And if we notice this and listen to the patient, then we might realise we have to move in a different direction.”*

*MST (interview 3) “ to me, It is a cultural thing. In todays’specialty training we are focussed on becoming a higly specialised specialist. For a big part of our training we are not thinking about the role of the GP and the fact that the patient will consultate their GP with questions the next day. So early in training we need to be made aware that when we see a patient we are only a small part of their care trajectory and we are working to get the patient as quickly at home as possible. And when we realise that, you feel the need to make sure this can be made possible, and you feel the need to collaborate.”*

#### Presence of role models

Participants felt that a collaborative culture in the workplace facilitates learning collaboration. Several participants stressed the importance of supervisors as role models, but mentioned at the same time that collaboration is often not recognised as an important quality of a doctor in practice. Participants described role models as people who give examples of exemplary PSCC which they can observe and who help creating a collaborative culture.

*ED (interview 2): Role modelling. Role modelling. Show how it should be done. Everything in the training programme needs to be shown the right way….*

*MS (interview 1)…………. there are role models I think. They are not very visible. When you ask: who do I need to do research, you get a quick answer: you have to go to him or her. But when you ask about who should learn me how to be a good collaborator? In surgery they will feel it is a “soft question”…*.

Participants also mentioned that ambassadors, for example a trainee and a supervisor together, could play a part in creating a culture that facilitates PSCC.

*MST (interview 2): “I think, that some sort of ambassador function, works well sometimes. You cannot really tell up front if it will work, or not, that depends on the resistance you get. But if you entitle it as an ambassador, thus that you are expected to carry out your task, then you can get actively involved in morning and evening reports, or other moments*.

Hence, when implementing an intervention for learning PSCC attention should be given towards the presence of role models or ambassadors to increase the chance of success.

#### The workplace provides time to learn PSCC

Both primary and secondary care trainees experience a lack of time due to work pressure and other priorities as a barrier to engage in a learning dialogue. They feel a need for dedicated time to learn PSCC in daily practice. A strategy to overcome this barrier is reserving time to engage in PSCC in the daily work schedule, not only for trainees, but for supervisors as well.

*MST (interview 1): “Time……. I think… So, if the training programme, would reserve, just a little bit but some dedicated time for it (visit a GP practice) than you show as a supervisor that you judge it (PSCC) as important.”*

*MS (interview 3) “digital consultation is a nice way of collaboration, I think. And when this is paid for then. so then you could have 10 consultations from GPs at the end of the day, but you do have two hours’ time for it”*.

Participants mentioned that it could help to place interventions in existing primary-secondary care contacts like consultation, triage or internships of GPTs in the hospitals as opportunities to discuss collaboration.

*MS (interview 2) …………………………… We need to look at the contacts between primary and secondary care that already exist, or interdisciplinary organised education. I think that is the way to go. GPT: I agree*.

#### Formalise learning PSCC in curricula

Another way to reserve dedicated time for learning PSCC is to include and describe learning goals for this collaboration in the speciality training curricula. In the current post graduate curricula PSCC is not included explicitly, no learning objectives are attached to PSCC and no assessment of this collaboration happens in daily practice. For supervisors it would be easier to create this time, if it were an explicit part of the training programmes.

*MS (interview 1) “It is about defining learning goals and make sure that this is judged as important enough to make it an obligatory part of the curricula. That’s it, then it will happen.”*

*MS (interview 3) “That is something we need to pay attention to in our medical specialty training programmes. That contact with and involvement of the GP during admission is part of good medical practice. And that you will be judged on that.”*

Our participants felt that supervisors should obligate their trainees to attend and engage in interventions to learn collaboration otherwise only trainees who like the subject would attend.

*GPT (interview 1): “So, I do not know how you guys feel about it, but I am not inclined to do it [ask for feedback eds.], when I do not have to do it.”*

*MST (interview 2): “I was just about to say that, because it might sound childish, but you know who will attend that intraprofessional education programme.” MST2: “It has to be obligatory.”*

#### Create a safe learning environment

One of the conditions to engage in a learning dialogue is that the environment where trainees learn is a safe one. Participants describe this as an environment with colleagues and educators who actively invite to reflect on collaboration. Furthermore, trainees should experience no hierarchy between GPs and MSs. This was specifically named in relation to using the GP trainees internships in secondary care as opportunities to discuss PSCC. Participants felt it was hard for a GP trainee to focus on collaboration and give feedback on this collaboration without being actively invited to do so in the secondary care environment.

*GPT (interview 2): It would be easier to give my viewpoint as a GP in training on PSCC when I would be actively invited to do so…. I would feel a high threshold to comment on PSCC in a new environment where everybody knows each other. But if they would ask me do to so… then…. ED:.so asking for feedback should have more attention? MST”: mutuality at least, the focus should no only be: we want to teach you something, but also what can we learn from you. We really want to know.*

In general, participants mentioned that knowing their collaboration partner personally would lower the threshold to contact them and engage in a dialogue. However, the number of trainees is high, and almost every day people start or end their training, so it is impossible to know everyone personally. The threshold could be lowered, when involved institutions organise days that trainees come together, both formal and informal.

### Updated literature search 2017–2023

We found five articles that met our inclusion criteria [44–48]. The preliminary design principles from our literature research were recognisable in more recent literature. However, in three articles ([44–46] an important reason for success of the interventions was meeting one-another. The importance of interaction was emphasized in previous articles (codes as face-to-face, interactive design, small team interventions) but not as explicitly mentioned as in these last articles. This is in line with the findings of our focus groups in which interaction is named as key for learning PSCC.

**Table 3:** Design principles for interventions to learn PSCC in postgraduate training programmes

Design principle	Interaction: Engage in a learning dialogue	Facilitate that the learning dialogue concerns collaboration	Create a workplace that facilitates engagement in a learning dialogue
**Description**	Learning PSCC is only possible through interaction with the collaboration partner (in training). Interventions should focus on interaction.	PSCC interventions should facilitate that the dialogue is about knowledge and skills needed for this collaboration instead of about pure medical content.	Without support from and changes in the work context interventions with the aim to learn PSCC will not succeed. This means that in development and implementation of interventions the workplace should be taken into account as a variable that affects learning PSCC.
**Subcategories**	Not applicable	Not applicable	1. Intervention emphasises the urge for PSCC and is based on daily practice 2. The presence of role models 3. The workplace provides time to learn PSCC 4. Learning PSCC is formalised in curricula 5. Create a safe learning environment

## Discussion

### Summary

In our research we defined three draft design principles with subcategories to learn PSCC in postgraduate training programmes: Interventions should focus on interaction between GP trainees and MS trainees, so they can engage in a learning dialogue. Interventions facilitate that the dialogue is about collaboration and interventions are placed in a workplace that facilitates engagement in a learning dialogue. Within this last design principle we defined 5 subcategories that could support changes in the work context: emphasis on the urge for PSCC and recognisability in daily practice, presence of role models, dedicated time to learn PSCC, formalisation of learning PSCC in curricula and a safe learning environment. The design principles we found in our literature review came up spontaneously in the group interviews, or were recognisable when we asked sounding board group participants about them. Stakeholders placed the design principles found in literature in the context of learning PSCC and that of the post graduate training programmes. This way they formulated more practical recommendations and made design principles more concrete.

### Comparison with literature

Learning PSCC is an example of learning at boundaries, in this case boundaries of two different care settings. To be able to learn PSCC boundaries should be crossed. One of the manners in which boundaries can be crossed is by boundary interactions: communication and collaboration between people across boundaries [49]. In our study interaction is seen as essential to learn PSCC. Akkerman et al. [50] described four types of learning related to boundary crossing: 1)Identification: boundary crossing can lead to the identification of the intersecting practices, whereby the nature of practices is (re)defined in light of one another. 2)Coordination: boundary crossing can lead to processes of coordination of both practices to make transitions smoother. 3)Reflection: learning to look differently at one practice by taking on the perspective of the other practice, and 4)transformation: boundary crossing can lead to changes in practices or even the creation of a new in-between practice [50]. Similarities can be seen in our research. Our participants mentioned that interaction should lead to knowledge on each other’s roles, expertise and contexts (identification), to collaboration agreements (coordination) and reflection on collaboration. When we look at the learning potentials boundary crossing holds and especially at coordination and transformation, these are not only important for individuals to learn PSCC but for the work context as well. This might help to increase the support from the work context to learn PSCC.

Looman et al. investigated the opportunities and barriers for learning intraPC in hospital placements of primary care trainees. They found that IntraPC is not learned spontaneously during hospital placements. One of main themes found was the work environment. The authors stated that learning intraPC is only possible when a safe work-learning climate and significant practicalities are secured. Looman et al. concluded that learning intraPC is promoted when there is a collaborative culture (with not too much hierarchy), dedicated time and goal setting for intraPC and support from the MS on the ward and GP teachers during release days [51]. This is in line with our findings.

Griffin et al. explored an integrative postgraduate curriculum for general practitioners and paediatricians in training. Participants stressed the importance of using the patient journey as a motivation to engage in integrated care. They also mentioned the value of mentors, a GP or paediatrician with expertise in integrated care, who were role models in integrated care and inspired participants [52]. In our focus groups presence of role models that show exemplary collaboration was named as a facilitator for learning PSCC. Furthermore participants saw a role for ambassadors to inspire and remind doctors of the importance of good PSCC.

Meijer et al. investigated the learning potential of making collaboration agreements between GPs and specialists. They found that participation helped to resolve contradictions and created opportunities to learn from each other and to find pleasure in work. Participants said that the most important outcome was getting to know one another and understanding each other’s daily practice. However dissemination of agreements in daily practice among non-participating professionals was difficult [53]. This, again, shows the importance of the work context in learning and delivering PSCC.

Comparing our research with literature on key factors for interprofessional (between different professionals) educational (IPE) interventions, we find similarities. Reeves et al. described that learning in a context that reflects the students’ current or future practice is important for effective learning. Resources, as space, time, attributes and funds need to be sufficient. Related to the importance of role models, teachers need to be qualified and trained for IPE facilitation [54, 55]. Negatively valued in IPE is the existence of professional hierarchies [54, 56–58]. In our research a safe learning environment without too much hierarchy was named as important to learn PSCC.

### Strengths and limitations

In our research we tried to answer a relevant practice-based question. Using a mixed methods approach gave us the opportunity to combine published knowledge with insights from stakeholders. As far as we know this is the first article giving an overview of design principles of intraprofessional educational interventions from literature. We used strictly formulated inclusion criteria, which helped to improve rigour. However, the number of included articles was low. We may have missed relevant data; we only had access to published literature, leaving university documents and grey-literature out [26]. Subsequently we conducted group interviews. Group interviews were highly suitable for gaining more insight into learning PSCC in the specific context of postgraduate training design as we spoke to stakeholders directly. A heterogenous group composition led to direct feedback on different perspectives. However, a disadvantage of a diverse group composition is the possibility of power imbalances and lack of respect for differing opinions. We did not experience this, but this does not mean that our participants were not hampered by power imbalance. Furthermore, in our group interviews there is a selection bias. We had a highly motivated group of participants not afraid to share their opinions. This might have led to fewer barriers and more opportunities mentioned for learning PSCC. Pilots and subsequent evaluation can help to gain more insight into design principles for a less selective population.

In qualitative research reflexivity is an important issue. All researchers were doctors (in training) with their own experiences and ideas on (learning) PSCC. Furthermore they were all motivated to implement learning PSCC. These characteristics of the researchers could have influenced analysis and the results. We did make sure the different perspectives were represented within the team: the primary and secondary care perspective, and the learning perspective as one of the researchers was also trained as an educationalist.

### Practice and future research

In order to make an intervention work we have to involve the work context. In a work context with high demands our participants stressed the importance of looking for contacts between primary and secondary care that already take place. It is necessary to link learning goals to these contacts and create time for feedback and reflection. These contacts can differ per trainee and per specialty, so it is probably not one intervention that will do the trick. Although learning moments should be sought on the workplace, support from education and curricula developers is needed to create time to engage in and reflect on learning PSCC.

In our research we determined design principles based on literature and group interviews with stakeholders. Following steps in DBR would be to design interventions, evaluate them and adjust design principles when appropriate [19, 21, 23]. Realist methods could help to identify which aspects of an intervention or context works for whom and why [25–27].

Furthermore, attention is needed to measurement of the effects of interventions: effects on collaborative behaviour, on PSCC and ultimately on patient care. Knowledge on instruments for measurement of PSCC is lacking [59].

If we compare our findings with research concerning IPE we see several similarities. We have witnessed these similarities between intraprofessional education (intraPE) and IPE before [44]. When designing intraPE, the use of evidence from IPE research could be useful since there is much more knowledge published in this field than in that of intraPE. In future DBR with a focus on learning PSCC, we suggest to involve stakeholders in design, implementation and evaluation of intraPE and have them use knowledge from research on both intraPE and IPE.

## Conclusions

In this multi-method study we formulated design principles for interventions in postgraduate training programmes with the aim to learn PSCC. Interaction is key in learning PSCC. This interaction should concern collaborative issues. Furthermore, it is essential to include the workplace in the intervention and make adjacent changes in the workplace when implementing an intervention to learn PSCC. The knowledge gathered in this study can be used to design interventions for learning PSCC. Evaluation of these interventions is needed to acquire more knowledge and adjust design principles when necessary.

## Electronic supplementary material

Below is the link to the electronic supplementary material.

Supplementary Material 1

Supplementary Material 2

Supplementary Material 3

## Data Availability

The datasets generated and analysed during the current study are not publicly available due to privacy concerns for the participants. They are available from the corresponding author on reasonable request.
