# Supplementary material for: Learning collaboration at the primary-secondary care interface: a dual-method study to define design principles for interventions in postgraduate training programmes
Source: BMC Med Educ. 2023 May 3;23:308. doi: 10.1186/s12909-023-04254-9 (PMC10158135; doi:10.1186/s12909-023-04254-9)
Supplement: Supplementary file 1 — Supplementary Material 1 [file 12909_2023_4254_MOESM1_ESM.docx]

Appendix 1 search strategy

| **Pubmed** |
| --- |
| ‘‘collaboration AND intraprofessional AND education AND primary care OR general practitioners, AND secondary care OR specialists, AND postgraduate'' |
| '((((("Education, Medical, Graduate"[Mesh] AND "Education, Medical, Continuing"[Mesh]) OR ( "Education, Medical/methods"[Mesh] OR "Education, Medical/organization and administration"[Mesh] ))) AND (((("Medical Staff, Hospital"[Mesh]) OR "Hospitalists"[Mesh]) OR "Hospital Medicine"[Mesh]) AND "Physicians"[Mesh])) AND ((((("Physicians, Family"[Mesh]) OR "Family Practice"[Mesh]) OR "General Practice"[Mesh]) OR "General Practitioners"[Mesh]) OR "Primary Health Care"[Mesh])) AND (("Intersectoral Collaboration"[Mesh]) OR "Global Health"[Mesh])'' |
| 'intraprofessional collaboration AND education'' |
| '((((((("Education, Medical, Graduate"[Mesh]) OR ("Internship and Residency"[Mesh]) AND "Education, Medical, Continuing"[Mesh]) OR ( "Education, Medical/methods"[Mesh] OR "Education, Medical/organization and administration"[Mesh] ))) AND (((("Medical Staff, Hospital"[Mesh]) OR "Hospitalists"[Mesh]) OR "Hospital Medicine"[Mesh]) AND "Physicians"[Mesh])) AND ((((("Physicians, Family"[Mesh]) OR "Family Practice"[Mesh]) OR "General Practice"[Mesh]) OR "General Practitioners"[Mesh]) OR "Primary Health Care"[Mesh])) AND (("Intersectoral Collaboration"[Mesh])'' |
| ''''((((("Education, Medical, Graduate"[Mesh] AND "Education, Medical, Continuing"[Mesh]) OR ( "Education, Medical/methods"[Mesh] OR "Education, Medical/organization and administration"[Mesh] ))) AND (((("Medical Staff, Hospital"[Mesh]) OR "Hospitalists"[Mesh]) OR "Hospital Medicine"[Mesh]) AND "Physicians"[Mesh])) AND ((((("Physicians, Family"[Mesh]) OR "Family Practice"[Mesh]) OR "General Practice"[Mesh]) OR "General Practitioners"[Mesh]) OR "Primary Health Care"[Mesh])) AND (("Intersectoral Collaboration"[Mesh]) OR "Global Health"[Mesh])'' |
| '(((((((("Education, Medical, Graduate"[Mesh]) OR ("Internship and Residency"[Mesh]) AND "Education, Medical, Continuing"[Mesh]) OR ( "Education, Medical/methods"[Mesh] OR "Education, Medical/organization and administration"[Mesh] ))) AND (((("Medical Staff, Hospital"[Mesh]) OR "Hospitalists"[Mesh]) OR "Hospital Medicine"[Mesh]) AND "Physicians"[Mesh])) AND ((((("Physicians, Family"[Mesh]) OR "Family Practice"[Mesh]) OR "General Practice"[Mesh]) OR "General Practitioners"[Mesh]) OR "Primary Health Care"[Mesh])) AND ((("Cooperative Behavior"[Mesh]) OR ("Intersectoral Collaboration"[Mesh]) OR ("Global Health"[Mesh])))'' |
| ''((((((("Education, Medical, Graduate"[Mesh]) OR ("Internship and Residency"[Mesh]) AND "Education, Medical, Continuing"[Mesh]) OR ( "Education, Medical/methods"[Mesh] OR "Education, Medical/organization and administration"[Mesh] ))) AND (((("Medical Staff, Hospital"[Mesh]) OR "Hospitalists"[Mesh]) OR "Hospital Medicine"[Mesh]) AND "Physicians"[Mesh])) AND ((((("Physicians, Family"[Mesh]) OR "Family Practice"[Mesh]) OR "General Practice"[Mesh]) OR "General Practitioners"[Mesh]) OR "Primary Health Care"[Mesh])) AND (("Intersectoral Collaboration"[Mesh]) '' |
| 'intraprofessional education'' |
| 'intraprofessional learning'' |
| 'intraprofessional collaboration AND education'' |
| **Cochrane Library search** filter: Publication Year from 2012 to 2017 |
| 'intraprofessional AND education'' |
| 'intraprofessional'' |
| 'collaboration AND intervention'' |
| 'collaboration AND education'' |
| **CINAHL** filter: Published date 2012 - 2017, Exclude MEDLINE records |
| 'intraprofessional collaboration AND intraprofessional education'' AND (''postgraduate OR residents")'' |
| **PsychINFO**  filter: Publication Year from 2012 to current |
| (Intraprofessional collaboration and education and (graduate physicians or residents)).mp. [mp=title, abstract, heading word, table of contents, key concepts, original title, tests & measures]'' |
| '(intraprofessional education and (postgraduates or residents)).mp. [mp=title, abstract, heading word, table of contents, key concepts, original title, tests & measures]'' |
| ('collaboration and intervention'').mp. [mp=title, abstract, heading word, table of contents, key concepts, original title, tests & measures]'' |
| ('collaboration and intervention and (postgraduates or residents)'').mp. [mp=title, abstract, heading word, table of contents, key concepts, original title, tests & measures]'' |
| ''collaboration and intervention'').mp. [mp=title, abstract, heading word, table of contents, key concepts, original title, tests & measures]'' |
| ('collaboration and intervention and (postgraduates or residents)'').mp. [mp=title, abstract, heading word, table of contents, key concepts, original title, tests & measures]'' |
| **ERIC**  filter: Publication Year from 2012 to current |
| '(Intraprofessional collaboration and education and (graduate physicians or residents)).mp. [mp=abstract, title, heading word, identifiers]'' |
| '(collaboration and intervention and (postgraduates or residents) and education).mp. [mp=abstract, title, heading word, identifiers]'' |
| '(collaboration and intervention and education).mp. [mp=abstract, title, heading word, identifiers]'' |
