# Supplementary material for: Learning collaboration at the primary-secondary care interface: a dual-method study to define design principles for interventions in postgraduate training programmes
Source: BMC Med Educ. 2023 May 3;23:308. doi: 10.1186/s12909-023-04254-9 (PMC10158135; doi:10.1186/s12909-023-04254-9)
Supplement: Supplementary file 2 — Supplementary Material 2 [file 12909_2023_4254_MOESM2_ESM.docx]

**Appendix 2: topic guide**

**Introduction**

What is your name? In which specialty do you work and what is your role?

*We will provide the participants with an explanation on the goal of this focus group session and the desired learning goals of the intervention based on previous research.*

**Opening question**

We would like to ask everyone to write ideas on learning collaboration on post-its during the following three minutes. This does not have to be healthcare specific. Think about how you learned to collaborate.

**Transition question**

Now look at all the ideas you wrote down and think about learning primary-secondary care collaboration in the postgraduate training programmes, which do you think have the most potential in this setting? Please place them on the whiteboard based on potential, the most potential at the left side, and the least potential on the right side.

*We will work from the described ideas. We will ask participants what is needed for making suggested ideas a success in the context of the postgraduate training programmes. The following questions will be used to explore possibilities.*

**Key questions**

What makes the suggested learning opportunity successful? Think about:

Guidance (Supervisors, teachers) Other participants? On the job, intertwined in daily practice? Off the job? Which elements do you value most?

What is necessary in realisation of the discussed idea

Opportunities during the training programmes?

Barriers? What could help to overcome these barriers?

Where and when in the training programme should the intervention take place and how can we realise that?

What would make you engage in an intervention/learning opportunity? As a trainee, supervisor, educator or developer?

How do you see your own role in learning collaboration?

*During a break the focus group leader and the assistant will check the found design principles in literature and will determine if they were discussed upon or if they should be checked after the break.*

In literature we found design principles for learning intraprofessional collaboration. We would like to hear your opinion on the role of these principles in learning primary-secondary collaboration in the postgraduate training programmes

- Personalised education: the intervention should be adjusted to the participants (think about form, content and entrance level). What would that mean for an intervention in the postgraduate training programmes?
- Work process involvement: what would help making the link to daily practice?

Furthermore there are several barriers in the workplace described, think about logistics. What are preconditions in this field for the success of interventions?

- Participatory design: giving participants an active role during an intervention and/or in developing the intervention seems to increase the change of success of an intervention. How do you feel about this?

Active role for participants during the intervention (giving and receiving feedback, perspective taking, small groups)

Active role in development or adjustment of an intervention.

- Role models: In literature the importance of good guidance, or role models is stressed. What are, in your opinion, criteria supervisors of an intervention should meet?

**Ending**

Does anyone want to add something that has not passed the discussion yet?

*Summarise and check summary with participants, give them a chance to add anything.*
