# Supplementary material for: Learning collaboration at the primary-secondary care interface: a dual-method study to define design principles for interventions in postgraduate training programmes
Source: BMC Med Educ. 2023 May 3;23:308. doi: 10.1186/s12909-023-04254-9 (PMC10158135; doi:10.1186/s12909-023-04254-9)
Supplement: Supplementary file 3 — Supplementary Material 3 [file 12909_2023_4254_MOESM3_ESM.docx]

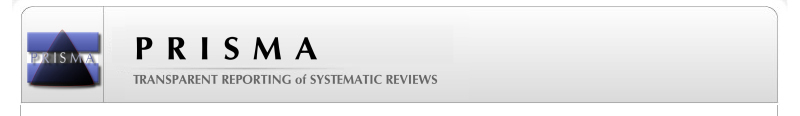
**PRISMA 2009 Flow Diagram**

Additional records identified through other sources (reference screening, reversed citing) (RL)
(n =56)

Full-text articles assessed for eligibility (RL and MJ)
(n =41)

Records screened, abstract and title (RL)
(n = 922)

Full-text articles excluded, with reasons
(n =33)

Records after duplicates removed
(n =922)

Studies included
(n =8)

Records identified through database searching 2012-may 2017 (RL)
(n =890)

Records excluded
(n =865)

## Identification

## Eligibility

## Included

## Screening
